# Ultrasound and laser-promoted dual-gas nano-generator for combined photothermal and immune tumor therapy

**DOI:** 10.3389/fbioe.2022.1005520

**Published:** 2022-09-13

**Authors:** XinYu Li, Yong Gao, XinZheng Liu, XiaoQian Hu, YunMeng Li, JunXi Sun, PingYu Wang, Hongkai Wu, HaeWon Kim, Murugan Ramalingam, ShuYang Xie, RanRan Wang

**Affiliations:** ^1^ Institute of Rehabilitation Medicine, School of Rehabilitation Medicine, Binzhou Medical University, Yantai, China; ^2^ Shandong Laboratory of Advanced Materials and Green Manufacturing, Yantai, China; ^3^ Binzhou Medical University Hospital, Binzhou, China; ^4^ Key Laboratory of Tumor Molecular Biology, Binzhou Medical University, Yantai, China; ^5^ Department of Chemistry, The Hong Kong University of Science and Technology, Hong Kong, China; ^6^ Institute of Tissue Regeneration Engineering, Dankook University, Cheonan, Korea; ^7^ Department of Nanobiomedical Science, BK21 NBM Global Research Center for Regenerative Medicine, Dankook University, Cheonan, Korea; ^8^ Mechanobiology Dental Medicine Research Center, Dankook University, Cheonan, Korea; ^9^ School of Basic Medical Sciences, Chengdu University, Chengdu, China

**Keywords:** CaCO_3_-PDA-MnO_2_ nanoparticles, dual-gas nano-generator, ultrasound and laser, photothermal, immunotherapy

## Abstract

The combination of photothermal therapy (PTT) and immune tumor therapy has emerged as a promising avenue for cancer treatment. However, the insufficient immune response caused by inefficient immunogenic cell death (ICD) inducers and thermal resistance, immunosuppression, and immune escape resulting from the hypoxic microenvironment of solid tumors severely limit its efficacy. Herein, we report an ultrasound and laser-promoted dual-gas nano-generator (calcium carbonate-polydopamine-manganese oxide nanoparticles, CPM NPs) for enhanced photothermal/immune tumor therapy through reprogramming tumor hypoxic microenvironment. In this system, CPM NPs undergo reactive decomposition in a moderately acidic tumor, resulting in the generation of calcium, manganese ions, carbon dioxide (CO_2_), and oxygen (O_2_). Calcium and manganese ions act as adjuvants that trigger an immune response. The cancer cell membrane rupture caused by sudden burst of bubbles (CO_2_ and O_2_) under ultrasound stimulation and the photothermal properties of PDA also contributed to the ICD effect. The generation of O_2_ alleviates tumor hypoxia and thus reduces hypoxia-induced heat resistance and immunosuppressive effects, thereby improving the therapeutic efficacy of combination PTT and immune therapy. The present study provides a novel approach for the fabrication of a safe and effective tumor treatment platform for future clinical applications.

## Introduction

Cancer is the second most common cause of death, seriously threatening human health (Liu J et al., 2019; Soerjomataram and Bray, 2021). Various treatment modalities such as surgery, radiotherapy, and chemotherapy have been used to eliminate tumors. However, the disadvantages of severe trauma, poor selectivity, and drug resistance greatly limit the application of these methods. Photothermal therapy (PTT) as an effective tumor treatment modality with non-invasive, highly targeted, low systemic toxicity and few side effects has got widespread attentions. PTT kills tumor cells by converting light energy into heat energy, and has received extensive attention (Li et al., 2018). However, in addition to effective tumor growth inhibition, avoiding tumor recurrence is crucial for effective tumor treatment. Immunotherapy, a rapidly developing therapeutic approach that inhibits tumor growth and prevents tumor recurrence and metastasis by activating the immune system, is considered to be one of the most promising therapeutic techniques for tumors. Since immunogenic cell death (ICD) plays a significant role in initiating antitumor immune responses, the development of potent ICD inducers is required to improve the efficacy of immunotherapy (Duan et al., 2019; Liu P et al., 2019; Chattopadhyay et al., 2020; Ding et al., 2020). Various ICD inducers have been reported, including radiation (Chao et al., 2018), photosensitizers (Deng et al., 2020), and chemotherapeutic drugs (Nam et al., 2018). However, X-rays and drug molecules can cause systemic toxicity and drug resistance, thereby increasing the risk of treatment. Therefore, development of mild and non-drug ICD inducers is required for safe and efficient immunotherapy. Currently, in addition to selecting efficient ICD inducers, remodeling the tumor hypoxic microenvironment is another important strategy to improve the immunotherapy efficiency (Hu et al., 2020; Li and Kataoka, 2021). Recently, Ca^2+^ has been reported to serve as an ICD inducer in immunotherapy. ICD effects are attributed to mitochondrial Ca^2+^ overload and the subsequent upregulation of the reactive oxygen species (ROS) levels (Zheng et al., 2021). Furthermore, exogenous ultrasound (US) stimuli can enhance Ca^2+^ overload by promoting Ca^2+^ influx from the extracellular fluid to the cytoplasm, resulting in enhanced ICD (Samal et al., 2000; Parvizi et al., 2002; Park et al., 2010). Another report showed that the sudden burst CO_2_ bubbles in the tumor microenvironment can also stimulate efficient immunogenicity by inducing membrane rupture of cancer cells (Jeon et al., 2022). Several studies have demonstrated that PTT can not only directly destroy tumor cells but also indirectly stimulate systemic immunity (Jiao et al., 2021; Yasothamani et al., 2021); therefore, the combined treatment using PTT and an ICD-inducer is anticipated to improve the efficiency of immunotherapy. Nevertheless, the hypoxic characteristics of tumors impair the efficiency of PTT and immunotherapy. For example, the hypoxic characteristics of tumor tissue leading to thermal resistance, reducing the efficiency of photothermal treatment (Meng et al., 2018; Wen et al., 2020; Dai et al., 2021; Lv et al., 2021). Moreover, hypoxia can lead to immune suppression and escape (Riera-Domingo et al., 2020; Zhou et al., 2020). MnO_2_ nanoparticles with tumor microenvironment-responsive degradation characteristics and catalase properties can react with excess H_2_O_2_ present in tumor tissue, producing O_2_ and Mn^2+^ to alleviate tumor hypoxia (Fan et al., 2015; Yang et al., 2017; Duan et al., 2020). In addition, recent studies have shown that MnO_2_ can be used as an immune adjuvant to trigger ICD by dual induction of Mn^2+^-triggered chemodynamic therapy (CDT) and glutathione (GSH)-depleting ferroptosis (Lv et al., 2020). CDT and ferroptosis can stimulate dying cancer cells to produce damage-associated molecular patterns (DAMPs), thereby eliciting ICD (Chen Y et al., 2019; Wen et al., 2019; Zhang et al., 2019).

In this study, we constructed ultrasonication- and laser-promoted dual-gas nanogenerators based on calcium carbonate (CaCO_3_), polydopamine (PDA), and manganese oxide (MnO_2_) nanoparticles for combined photothermal/immune tumor therapy. CaCO_3_ has the characteristics of accessibility, good biocompatibility, and pH sensitivity and can decompose at the tumor site, generating carbon dioxide (CO_2_) and Ca^2+^ (Feng et al., 2018; Yu et al., 2018; Sun et al., 2019; Wang et al., 2020). The inherent photothermal properties of PDA make it suitable for PTT (Wang et al., 2018; Chen Q et al., 2019; Li et al., 2020). MnO_2_ can decompose into oxygen and manganese ions in the tumor microenvironment. Therapeutic systems do not involve chemical drugs, preventing drug resistance. When delivered to the tumor site, the CPM NPs undergo reactive decomposition in a moderately acidic tumor microenvironment, resulting in the generation of Ca^2+^, Mn^2+^, CO_2_, and O_2_. The generated Ca^2+^ and Mn^2+^ act as immune adjuvants to trigger ICD. The cancer cell membrane rupture caused by sudden burst of CO_2_, O_2_, and the photothermal properties of PDA also contributed to ICD. The generation of O_2_ alleviates tumor hypoxia and thus reduces the hypoxia-induced heat resistance and immunosuppressive effects, thereby improving the therapeutic efficacy of PTT and immune therapy. In this treatment system, the combination of photothermal therapy and immunity can not only greatly inhibit tumor growth but also avoid tumor metastasis and secondary recurrence through immune effects. Finally, the tumor microenvironment-responsive degradation of the nanoparticles ensures their biosafety.

## Experimental details

### Materials

CaCl_2_ and NH_4_HCO_3_ were provided by Sinopharm Chemical Reagent Co., Ltd. (China). Dopamine was obtained from Sigma-Aldrich. Manganese chloride (MnCl_2_) was provided by J and K Chemical Co. Singlet oxygen sensor green reagent was purchased from Meilunbio. Sodium hydroxide (NaOH) was provided by Tianjin Yongda Chemical Reagent Co., Ltd. Calcein-AM/propidium iodide (PI) was purchased from SolarBio. RPMI-1640 medium was purchased from Thermo Fisher Scientific, Inc. Fluorescein isothiocyanate (FITC) was provided by Biological Technology Co., Ltd. Lyso-Tracker Red and Fluo-4 AM were purchased from KeyGen Biotech Co., Ltd. 5,5′,6,6′-Tetrachloro-1,1′,3,3′-tetraethylbenzimidazolyl-carbocyanine chloride (JC-1) and mitochondrial extraction kit were purchased from Solarbio.

### Characterization

The morphology and size of the CPM NPs were analyzed using a JEM-1400 transmission electron microscope (TEM, JEOL, Japan). The size and surface charge of the CaCO_3_-PDA (CP)and CPM NPs were analyzed using a Malvern Zetasizer Nano-ZS90 instrument (United Kindom). X-ray photoelectron spectroscopy (XPS) spectra were obtained using a Thermo Fisher Scientific ESCALAB 250Xi XPS system. The concentration of Ca^2+^ in the CPM NPs was determined using inductively coupled plasma mass spectrometry (ICP-MS, Jena, PlasmaQuant® MS). Cell uptake was measured using a confocal laser-scanning microscope (CLSM ZEISS LSM 780, Carl Zeiss, Jena, Germany).

### Cell culture and animal studies

4T1 cells were cultured in RPMI-1640 medium containing 10% fetal bovine serum. Then, the cells were placed in an incubator containing 5% CO_2_ at 37°C. Healthy female BALB/c mice aged 5–7 weeks were provided by GemPharmatech Co., Ltd. (Nanjing, China). All animals were euthanized by dislocation of cervical vertebra after the treatments.

### Synthesis of calcium carbonate-polydopamine-manganese oxide nanoparticles

CP NPs were prepared *via* a one-pot gas diffusion method following previously reported procedures with some modifications (Dong et al., 2018). Briefly, CaCI_2_ (150 mg) mixed with dopamine (10 mg) was fully dissolved in anhydrous ethanol (100 ml). Then, the samples were transferred into a flask with NH_4_HCO_3_ (5 g) and kept at 40°C for 24 h. Subsequently, CP NPs were obtained *via* centrifugation. To obtain CPM NPs, CP NPs (180 mg) were slowly dropped into an ethanol solution (30 ml) containing MnCl_2_ (360 mg). After stirring for 4 h, the Mn-ion-adsorbed CP nanoparticles were collected by centrifugal purification. Then, NaOH (1 M) was mixed with the solution by adjusting the pH to 11 to obtain CPM NPs.

### Oxygen and reactive oxygen species generation *in vitro*.

The oxygen probe was immersed in different solutions (100 μM H_2_O_2_, pH 7.4 + CPM, pH 6.5 + CPM, pH 7.4 + CPM+100 μM H_2_O_2_, pH 6.5 + CPM+100 μM H_2_O_2_, and CPM 1 mg ml^−1^) to determine the oxygen concentration. ROS levels were assessed using an ROS probe (reagent singlet oxygen sensor green, SOSG). SOSG methanol solution (10 μL, 2.5 M) was mixed into various solutions with CPM NPs (1 mg ml^−1^). Then, a 1,064 nm laser (1 W cm^−2^, 5 min) and/or US (1.0 MHz, 20% duty cycle, 0.5 W cm^−2^, 2 min) were applied to the mixture. Finally, a fluorescence spectrophotometer (Hitachi F-2700, Japan) was used to assess the SOSG signals of various groups.

### Investigation of Ca^2+^ Release from calcium carbonate-polydopamine-manganese oxide nanoparticles *in vitro*.

CPM NPs (1 mg) were immersed in PBS (10 ml) at 37°C and different pH (7.4, 6.5, and 5.5). Supernatants were collected from each sample at 0, 4, 8, 12, and 16 h. The amount of released Ca^2+^ was measured by Inductively coupled plasma mass spectrometry (ICP-MS). The release fractions of Ca^2+^ were calculated as the amount of released Ca^2+^ from CPM NPs divided by the total amount of Ca^2+^ in CPM NPs.

### Antitumor therapy *in vitro*

The antitumor effect and biocompatibility of the CPM NPs were determined using the MTT assay. 4T1 cells were co-cultured with CPM NPs at various concentrations for 12 h. The antitumor effect of CPM NPs was measured after the treatment with a 1,064 nm laser (1 W cm^−2^, 5 min) and/or US (1.0 MHz, 20% duty cycle, 0.5 W cm^−2^, 2 min). Thereafter, the cell survival rate was measured after adding a medium containing MTT and incubating for 4 h. For live/dead staining, CPM NPs were co-incubated with 4T1 cells for 12 h. Subsequently, the Calcein-AM/PI probe was added to the cell medium after the treatment with the laser and/or ultrasonication and was incubated for 30 min. Finally, images were obtained by CLSM. For apoptosis, CPM NPs (20 μg ml^−1^) were added to the 4T1 cells and cultured for 12 h. Thereafter, various treatments were applied to the cells. After 2 h, the cells were stained with annexin V-FITC/PI for flow cytometry detection.

### Detection of intracellular reactive oxygen species levels

Various conditions were detected using dichloro-dihydro-fluorescein diacetate (DCFH-DA) reagent (PBS, CPM, CPM + US, CPM + L, CPM + US + L, CPM: 20 μg ml^−1^, 1,064 nm laser: 1 W cm^−2^, 5 min and US: 1.0 MHz, 20% duty cycle, 0.5 W cm^−2^, 2 min) to generate intracellular ROS. The 4T1 cells plated on confocal dishes were co-incubated with CPM NPs for 12 h in the dark. Thereafter, the cells were treated by various treatments. Finally, CLSM was used to examine the fluorescence images.

### Lysosome escape

CPM NPs (labeled with FITC; 20 μg ml^−1^) were added to 4T1 cells and incubated for 0.5, 1, and 4 h. Then, the cells were labeled with Lyso-Tracker Red (1 μM). After 4 h, a 1,064 nm laser (1 W cm^−2^, 5 min) and/or US (1.0 MHz, 20% duty cycle, 0.5 W cm^−2^, 2 min) were applied to the cells, and 4% paraformaldehyde solution was added to fix the cells. After 10 min of fixation, DAPI was added to stain the cells. Fluorescence images were obtained by CLSM after 10 min.

### JC-1 Staining to Measure Mitochondrial Membrane Potential

CPM NPs (20 μg ml^−1^) were incubated with the 4T1 cells for 12 h. After various treatments, JC-1 (10 μg ml^−1^) was incubated with cells within 10 min. Subsequently, the mitochondrial membrane potentials were visualized using CLSM images.

### Mitochondrial swelling study

The 4T1 cells were incubated with CPM NPs (20 μg ml^−1^) for 12 h. After various treatments, the mitochondria of the 4T1 cells in various groups were extracted using a mitochondrial extraction kit, and mitochondrial swelling was analyzed by UV-Vis–NIR spectrophotometry to determine the optical intensity of the mitochondria at 540 nm.

### Intracellular Ca^2+^ detection in 4T1 cells

CPM NPs (20 μg ml^−1^) were cultured with 4T1 cells for 0, 1, and 4 h. Subsequently, Fluo-4 AM (1 μM) was used to label cells after incubation for 4 h. Then, a 1,064 nm laser (1 W cm^−2^, 5 min) and/or US (1.0 MHz, 20% duty cycle, 0.5 W cm^−2^, 2 min) were applied to the cells. Finally, fluorescence images were visualized by CLSM after co-incubation with Hoechst 33,342 for 10 min.

### Immunogenic cell death and dendritic cell maturation *in vitro*

To evaluate calreticulin (CRT) expression after various treatments, 4T1 cells were incubated with CPM NPs (20 μg ml^−1^) for 12 h. After a treatment with a 1,064 nm laser (1 W cm^−2^, 5 min) and/or US (1.0 MHz, 20% duty cycle, 0.5 W cm^−2^, 2 min), the 4T1 cells were incubated with CRT antibodies (1:100; Abcam, EPR3924) for 30 min. After washing three times with PBS, Cy5-labeled goat anti-mouse IgG (H + L) (1:400, Abbkine, A23320) was added to the cells and incubated at 37°C for 2 h. Finally, CLSM was used to visualize the cells after staining with DAPI for 15 min. The release of ATP and HMGB1 was examined using the Chemiluminescence ATP Determination Kit (Solarbio, BC0300) and the HMGB1 enzyme-linked immunosorbent assay (ELISA) Kit (CUSABIO, CSB-E08225M). Briefly, 4T1 cells were incubated with 20 μg ml^−1^ CPM NPs for 12 h. Then, after a treatment with a 1,064 nm laser (1 W cm^−2^, 5 min) and/or US (1.0 MHz, 20% duty cycle, 0.5 W cm^−2^, 2 min), the release level of HMGB1 in the cell culture medium was detected using an HMGB1 ELISA Kit. Similarly, the release of ATP was measured using an ATP ELISA kit. To determine DCs maturation, 4T1 cells were cultured with CPM NPs (20 μg ml^−1^) for 12 h. After a 1,064 nm laser (1 W cm^−2^, 5 min) and/or US (1.0 MHz, 20% duty cycle, 0.5 W cm^−2^, 2 min) treatment, the supernatant in the 6-well plate of the 4T1 cells was added to the 6-wall plates seeded with DC cells (derived from the bone marrow of BALB/c mice after stimulation with GM-CSF) and then cultivated for 24 h. The control group was cultured in a fresh medium. DCs were detected using flow cytometry after staining with anti-CD11c-PE (BioLegend, 117,308), anti-CD80-PE/Cy5.5 (Biolegend, 104,722), and anti-CD86-APC (BioLegend, 105,012).

### *In vivo* antitumor efficiency

When the primary tumor volume grew to approximately 80 mm^3^, BALB/c mice were randomly divided into five groups: PBS (100 μl), CPM (CPM: 100 μl, 1 mg ml^−1^), CPM + US (1.0 MHz, 20% duty cycle, 2 W cm^−2^, 2 min), CPM + L (1 W cm^−2^, 5 min), CPM + US + L, and were subcutaneously injected with four injections of CPM NPs or PBS on days 0, 2, 4, and 6. The temperature of the tumor area was recorded using an infrared thermal imager (FLIR E8). Tumor weight and volume were monitored on alternating days for 15 days. After treatment, the mice were euthanized by dislocation of cervical vertebra and all major organs and tissues were removed and stained with hematoxylin and eosin. To further investigate immune responses during photothermal and immune tumor therapy, tumor-draining lymph nodes, spleens, and tumors were collected after 15 days of treatment. To assess the maturity of DCs *in vivo*, single-cell suspensions obtained after lymph node grinding were stained with anti-CD11c-PE (BioLegend, 117,308), anti-CD80-PE/Cy5.5 (Biolegend, 104,722), and anti-CD86-APC (BioLegend, 105,012), and then were analyzed by flow cytometry. Additionally, to examine the infiltration of T lymphocytes *in vivo,* we harvested cells from the spleen and stained them with anti-CD8-FITC, anti-CD4-PE, and anti-CD3-APC for further flow cytometry analysis. Typical cytokines at the tumor sites were also analyzed. First, the supernatant was collected after centrifugation. ELISA kits were used to detect the cytokine IFN-γ, TNF-α, and granzyme B secretion levels.

### Statistical analysis

All experiments were repeated at least three times. Differences between groups were tested using Student’s *t*-test. Data were analyzed using the GraphPad Prism (version 5.01) software with significance levels set to the following probabilities: *p < 0.05, **p < 0.01, ***p < 0.001.

## Results and discussions

### Synthesis and characterization of the nanoparticles

**SCHEME 1:**
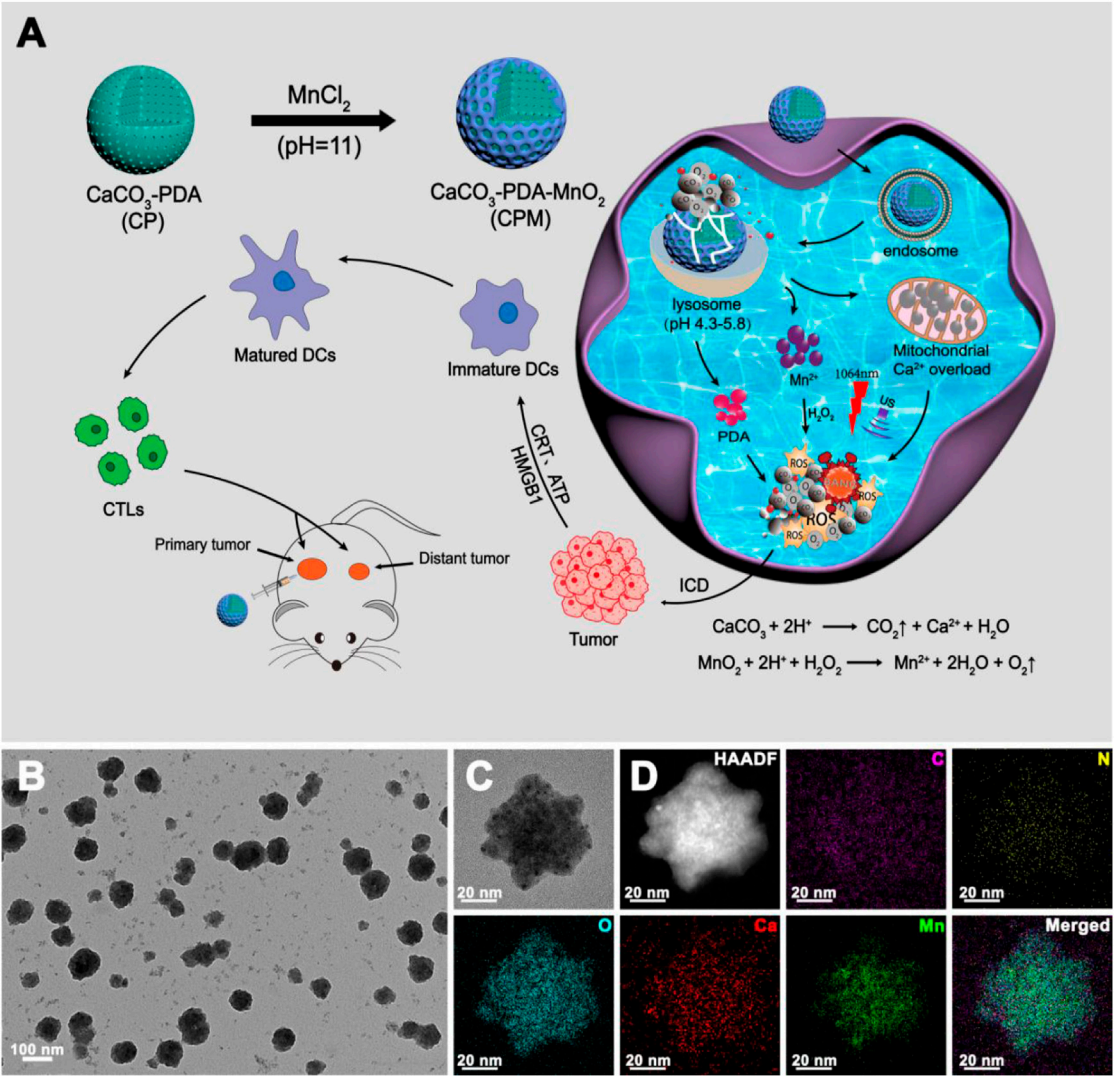
Schematic illustration and characterization **(A)** schematic illustration of the synthetic route of CPM NPs and its anti-tumor mechanism. **(B,C)** Transmission electron microscopy (TEM) image and **(D)** Element mappings of CPM NPs.

**FIGURE 1:**
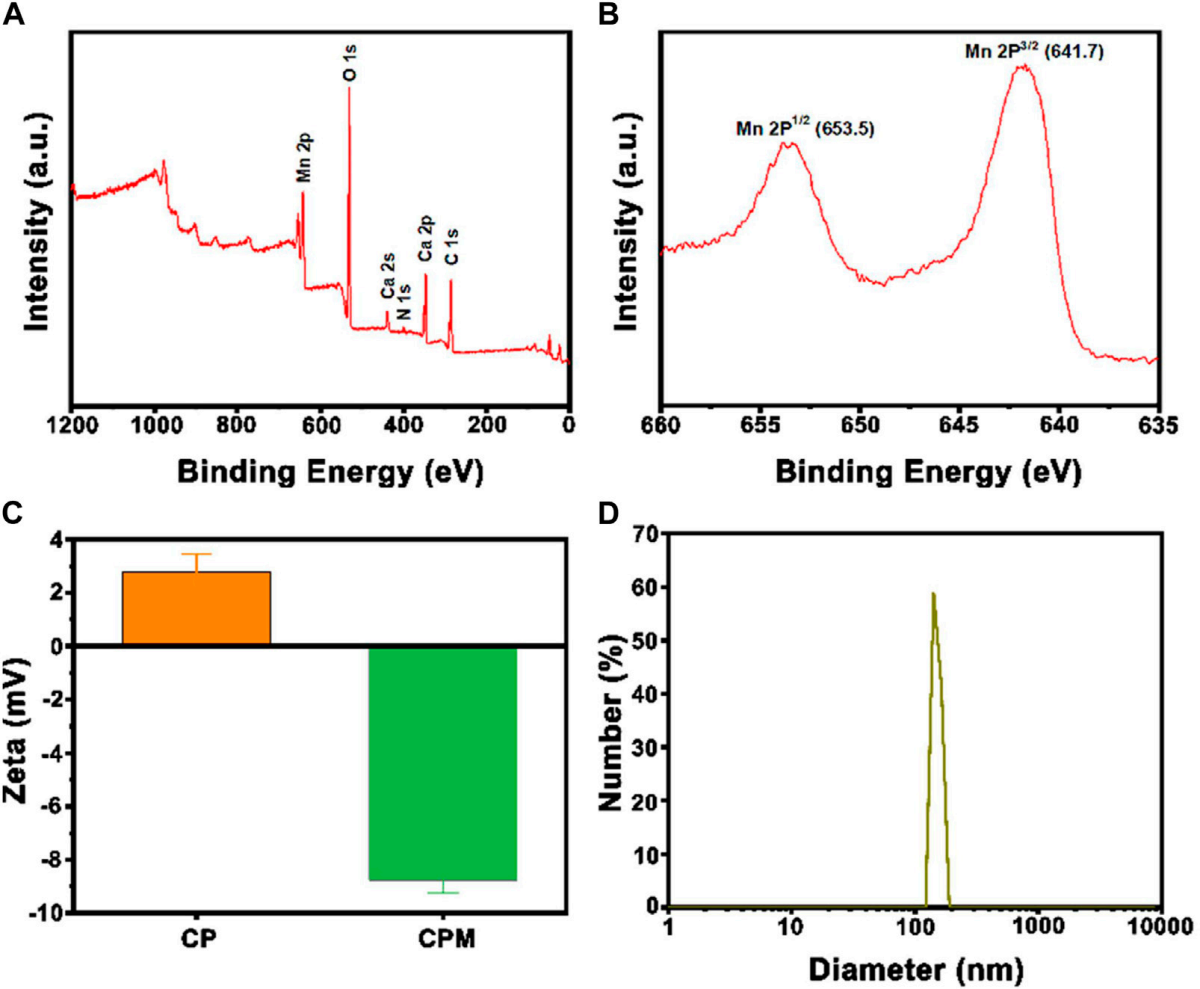
**(A)** XPS spectra of CPM NPs. **(B)** XPS high-resolution scans of Mn2p peaks in CPM NPs. **(C)** The average zeta potentials of CP NPs and CPM NPs. **(D)** Size distribution of CPM NPs.

The synthetic route of CPM NPs and their antitumor mechanism are illustrated in Scheme 1A. First, CaCO_3_-PDA nanoparticles (CP NPs) were synthesized by a typical one-pot gas diffusion method according to a previous report with minor modifications (Dong et al., 2018). Then, MnO_2_ was introduced to the surface of the CP NPs by the reaction of MnCl_2_ and NaOH to form CPM NPs. TEM was used to investigate the morphology and size of the CPM NPs. As shown in Schemes 1B,C, the CPM NPs with irregular spherical shapes exhibited a size of ∼150 nm. This size is suitable for cellular uptake. The corresponding elemental mapping demonstrated the homogeneous elemental distributions of C, O, N, Ca, and Mn in the CPM NPs (Scheme 1D). XPS analysis also confirmed the presence of C, N, O, Ca, and Mn in the CPM-NPs (Figure 1A **)**. Moreover, the characteristic peaks at 653.5 and 641.7 eV in the high-resolution Mn 2p spectrum correspond to the Mn (IV) 2p1/2 and Mn (IV) 2p3/2 spin-orbit peaks of MnO_2_, respectively (Figure 1B), illustrating the successful synthesis of CPM NPs (Chen et al., 2015; Zhao et al., 2015; Liu J et al., 2021). We also measured the zeta potentials of the CP and CPM NPs. As shown in Figure 1C, CPM NPs exhibited a lower average zeta potential (−8.76 mV) than CP NPs (2.79 mV). Negatively charged CPM NPs prolong blood circulation *in vivo*. To evaluate the dispersity of CPM NPs in water, dynamic light scattering (DLS) was used to detect the hydrodynamic diameter of the CPM NPs. As illustrated in Figure 1D, the CPM NPs showed an average hydrodynamic diameter of ∼150 nm, demonstrating the superior dispersibility of CPM NPs in water. We further investigated the colloidal stability of CPM NPs by immersing CPM NPs in various solutions. After 7 days of immersion in water, phosphate-buffered saline (PBS, pH 7.4), FBS, and Medium, the digital photograph of CPM NPs did not display any obvious precipitation (Supplementary Figure S1). Homoplastically, the hydrodynamic diameter of CPM NPs showed non-significant change after immersion in water for a week (Supplementary Figure S2). These results clearly revealed the excellent stability of CPM NPs. Notably, CPM NPs were stable in the presence of excess FBS protein, suggesting that CPM NPs can be stable in the blood stream (Hwang et al., 2008).

As the bubble generation ability of CPM NPs is crucial for this therapeutic system, we evaluated its bubble generation ability carefully under various conditions in the following study. Theoretically, in a simulated tumor environment with slightly acidic and high levels of hydrogen peroxide, CaCO_3_ nanoparticles can be decomposed to produce CO_2_ and Ca^2+^ by the following reaction: CaCO_3_+2H^+^→CO_2_+Ca^2+^+H_2_O. At the same time, MnO_2_ nanoparticles can be disintegrated to generate O_2_ and Mn^2+^ ions by the following reaction: MnO_2_+2H^+^+H_2_O_2_→Mn^2+^+2H_2_O + O_2_ (Sun et al., 2021). As shown in Supplementary Figure S3, both CP NPs and CPM NPs showed a certain bubble generating ability in pH 6.5 solutions with the existence of H_2_O_2_. The stimulation of laser or ultrasound promote the generation of bubbles both in CP NPs and CPM NPs. Obviously, the CPM + US + L + H_2_O_2_ group produced the largest number of bubbles compared to the other groups. Hence, it is reasonable to speculate that the dual gas production of O_2_ and CO_2_ in CPM NPs solutions greatly contributes to the large amounts of bubbles compared to CP NPs solution that only produces CO_2_.

**FIGURE 2:**
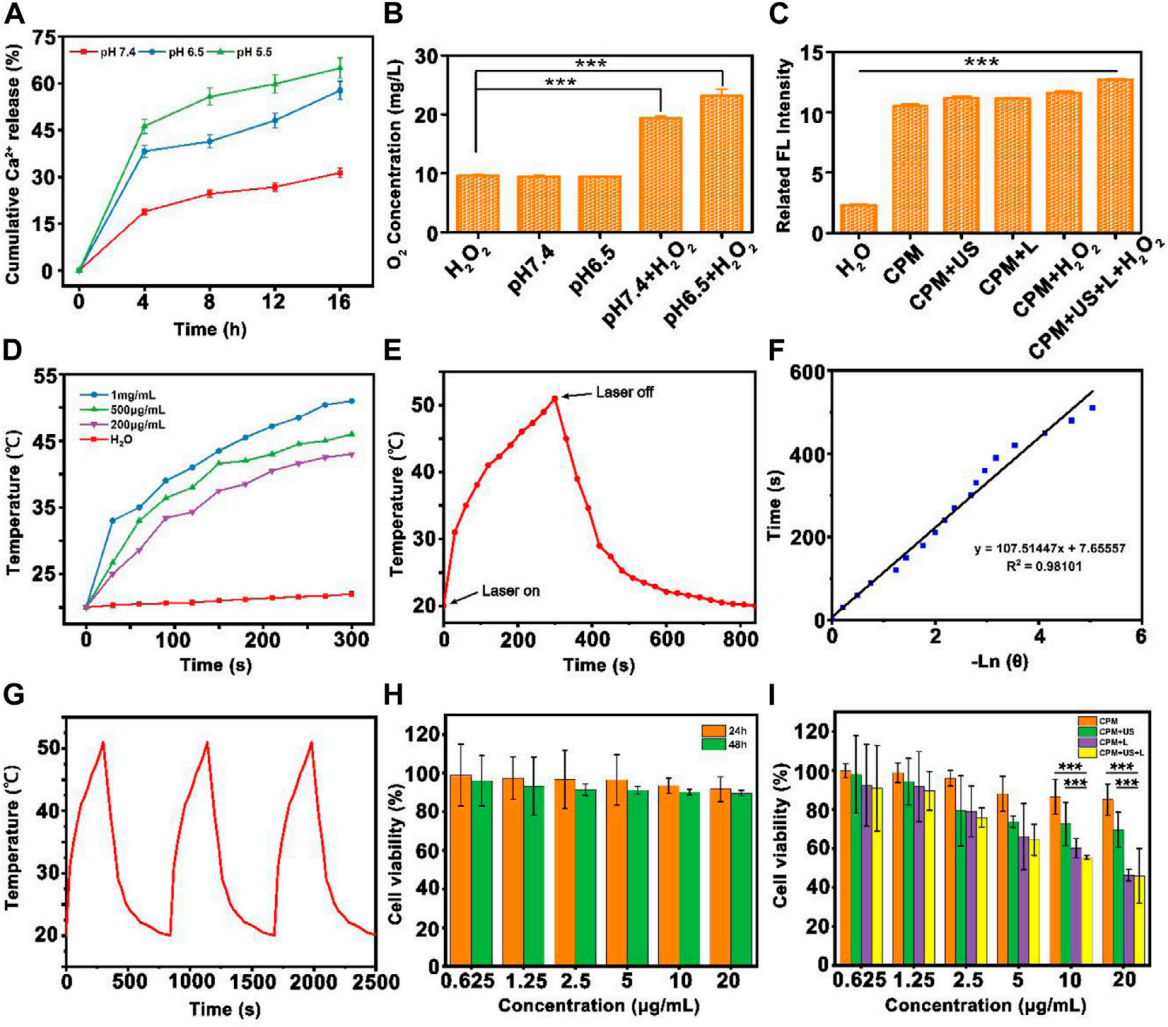
The physicochemical characterization of CPM NPs. **(A)** Time-dependent release profiles of Ca^2+^ from CPM NPs in PBS at different pH values 5.5, 6.5, and 7.4. The release percentages were calculated as the amount of release of Ca^2+^ from CPM NPs/the total amount of Ca^2+^ in CPM NPs. **(B)** The oxygen concentrations produced by CPM NPs at different pH values in H_2_O_2_ solution (100 μM). **(C)** SOSG fluorescence intensities of materials under different conditions. **(D)** Heating curves of different concentrations of CPM NPs under 1,064 nm laser irradiation. **(E)** Temperature record of CPM NPs (1 mg ml^−1^) under laser irradiation. **(F)** Linear-time data versus-ln (θ) obtained from the cooling period of NIR laser off. **(G)** The photothermal conversion cycling test of CPM NPs (1 mg ml^−1^) during three laser on/off cycles. **(H)** Viability of 293T after being treated with different concentrations of CPM NPs for 24 and 48 h. **(I)** Cell survival rate of 4T1 cells after being treated by different treatment groups.

Since pH-responsive Ca^2+^ release from CPM NPs is crucial for the calcium-overload-induced cancer immune therapy, we evaluated the Ca^2+^ release ability at various pH (5.5, 6.5, and 7.4). As shown in Figure 2A, time-related release behavior of Ca^2+^ from CPM NPs was observed. CPM NPs in the pH 5.5 solutions showed the highest calcium release rate (65%) compared to the pH 7.4 (31%) and pH 6.5 (58%) solutions. These results demonstrate the pH-dependent release behavior of CPM NPs. Therefore, CPM NPs are promising for Ca^2+^-induced immunotherapy.

As previously reported, MnO_2_ nanoparticles can trigger the decomposition of H_2_O_2_ to produce oxygen and Mn^2+^ by consuming H^+^ ions (Liu Y et al., 2019). Using a portable oxygen-dissolving apparatus, we further investigated the oxygen generation capability of CPM NPs at different pH with and without H_2_O_2_ (100 μM). A similar oxygen content of approximately 10 mg/L was observed in the pure H_2_O_2_ and PBS (pH 7.4) group (CPM NPs dissolved in pH 7.4 PBS solution) and the pH 6.5 group (and the CPM NPs dissolved in pH 6.5 PBS solution). When H_2_O_2_ was added, the pH 7.4 + H_2_O_2_ group exhibited significantly increased oxygen levels (∼20 mg/L). Compared to the other groups, the pH 6.5 + H_2_O_2_ group exhibited the highest oxygen content (∼24 mg/L) (Figure 2B). These results demonstrated the potential of CPM to eliminate tumor hypoxia. Then, the ROS generation capability of CPM NPs in pH 6.5 solution under various conditions was investigated. The fluorescence intensity was clearly increased in the presence of CPM NPs under various treatments compared to that in the pure H_2_O group (Figure 2C). However, the highest fluorescence intensity was obtained by the implementation of laser and ultrasound irradiation with H_2_O_2_. The above results show that laser and ultrasound irradiation can promote the production of ROS along with CPM NPs in the tumor microenvironment.

### *In vitro* photothermal effect of calcium carbonate-polydopamine-manganese oxide nanoparticles

As reported in previous studies, both PDA and MnO_2_ exhibit excellent photothermal conversion capability (Farokhi et al., 2019; Liu Y et al., 2021), which inspired us to evaluate the photothermal properties of the CPM NPs. We examined the heating curves of CPM NPs at different concentrations under 1,064 nm laser irradiation (1,064 nm, 1 W cm^−2^). The CPM NPs exhibited both time- and concentration-dependent photothermal heating (Figure 2D). After 15 min of laser stimulation, the temperature of the CPM NPs with a concentration of 1 mg ml^−1^ increased significantly from 20°C to 51°C while the temperature of the CPM NPs with a concentration of 200 μg ml^−1^ increased to 43°C. Subsequently, we calculated the photothermal conversion efficiency of CPM NPs to be 26.31% (Figures 2E,F). As depicted in Figure 2G, no apparent temperature decrease was observed during the 1,064 nm laser irradiation for three on/off cycles, indicating the excellent photothermal stability of the CPM NPs.

### Antitumor effects *in vitro*

To evaluate the combined antitumor photothermal and immunity effect *in vitro*, the cytotoxic effect of CPM NPs on tumor cells under various conditions was investigated. A low toxicity to normal cells is a necessary requirement for the application of CPM NPs in biological systems. We used normal human renal epithelial cells (293T) to evaluate the biosafety of CPM NPs by incubating the cells with different concentrations of CPM NPs for 24 or 48 h. It was found that the cell survival rate exceeded 90% even for incubation with the highest concentration (20 μg ml^−1^) for 48 h (Figure 2H), reflecting the excellent biocompatibility of CPM NPs. The antitumor effects of CPM NPs were further investigated. Various concentrations (0.625, 1.25, 2.5, 5, 10, and 20 μg ml^−1^) of CPM NPs were co-incubated with 4T1 cells and treated with 1,064 nm laser irradiation (1 W cm^−2^, 5 min) and US (1.0 MHz, 20% duty cycle, 0.5 W cm^−2^, 2 min). As shown in Figure 2I, the CPM group exhibited weak killing ability (85%) of 4T1 cells (20 μg ml^−1^) compared to the CPM + US (69.6%) and CPM + L groups (46.3%). The decreased cell viability of the CPM + US group may be attributed to the ultrasound-promoted calcium overload. Hyperthermia triggered by the photothermal effect of CPM NPs under laser irradiation also promoted cell death. The CPM + US and CPM + L groups exhibited concentration-dependent cell viability. As expected, the 4T1 cells treated with CPM + US + L showed the highest cell lethality (45.9%), indicating the most severe cell damage.

**FIGURE 3:**
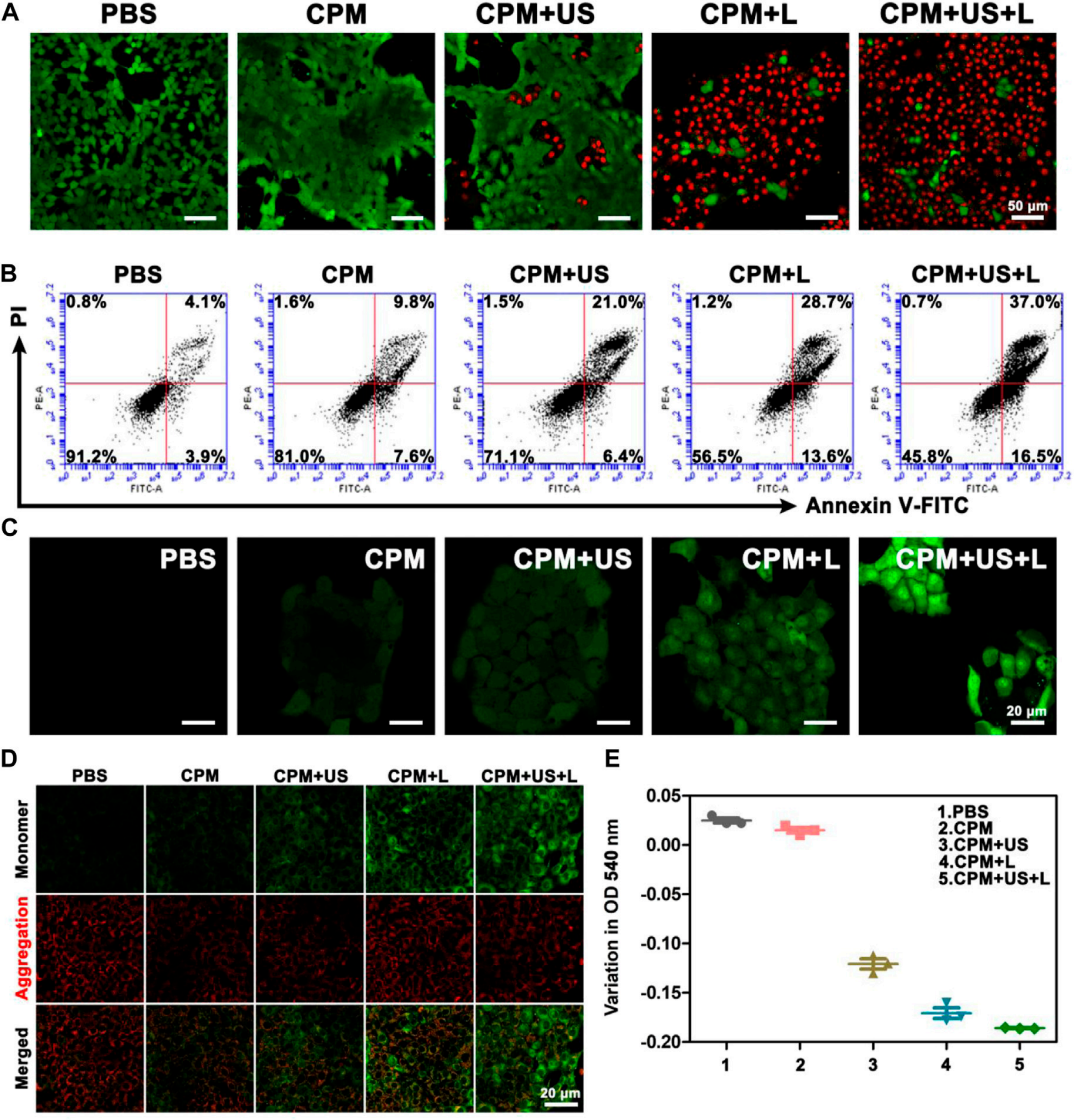
**(A)** Live/dead staining assay (green, live cells; red, dead cells) and **(B)** apoptosis analysis of 4T1 cells after various treatments. **(C)** Intracellular ROS detected by DCFH-DA probe. **(D)** CLSM images of 4T1 cells that received various treatments and then stained with JC-1 dye. **(E)** The optical density of 4T1 cells mitochondria at 540 nm after various treatments.

**FIGURE 4:**
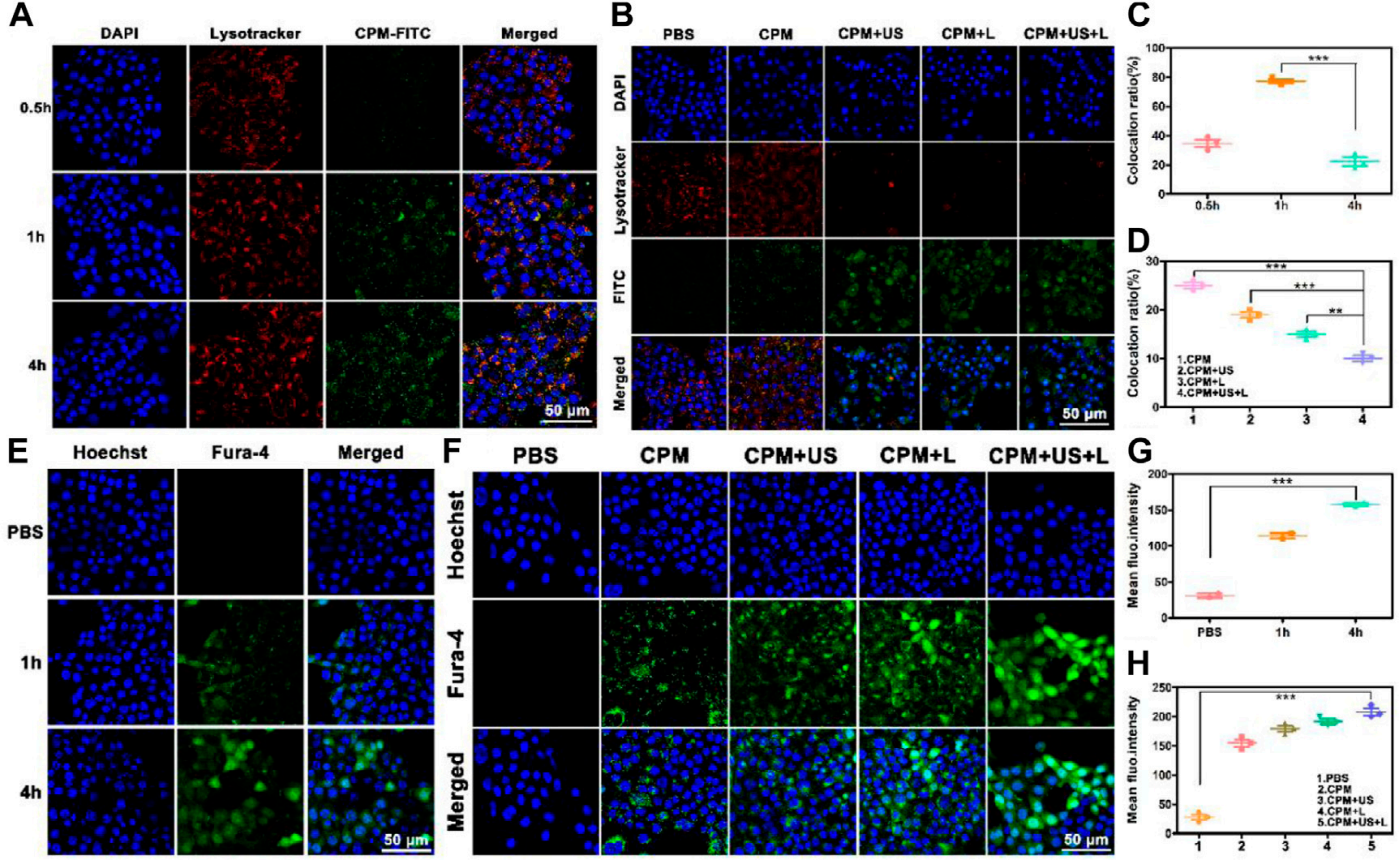
**(A)** The CLSM of CPM NPs (labeled with FITC, 20 μg ml^−1^) with lysosomes in 4T1 cells after incubation with CPM NPs for 0.5, 1, and 4 h, respectively. **(B)** The CLSM of CPM NPs (labeled with FITC, 20 μg ml^−1^) with lysosomes in 4T1 cells being various treatments. **(C)** The co-location ratio of CPM NPs with lysosomes in 4T1 cells after incubated with CPM for 0.5, 1, and 4 h, respectively. **(D)** The co-location ratio of CPM NPs with lysosomes in 4T1 cells after various treatments. **(E)** Confocal images of 4T1 cells incubated with CPM NPs (20 μg ml^−1^) at different time points and stained with an intracellular Ca^2+^ indicator, Fluo-4 AM (5 μM). **(F)** Confocal images of 4T1 cells being various treatments after incubated with CPM NPs (20 μg ml^−1^) for 4 h and stained with an intracellular Ca^2+^ indicator, Fluo-4 AM (5 μM). **(G)** Mean fluorescence intensity (MFI) of intracellular Ca^2+^ level in 4T1 cells. **(H)** MFI of intracellular Ca^2+^ level in 4T1 cells after various treatments.

To depict cell viability more intuitively, calcein-AM/PI was employed to conduct a living/dead assay by staining dead cells and living cells to red and green, respectively. As depicted in Figure 3A, only bright green fluorescence, representing live cells, appeared in the CPM and PBS groups, confirming the negligible killing effect. A few dead cells appeared in the CPM + US group, while more dead cells were observed in the CPM + L group, proving that the cytotoxic effect of photothermal therapy is better than that of ultrasound. As expected, the CPM + US + L group showed the highest number of dead cells compared to the other groups. This result was consistent with that of the MTT assay (Figure 2I). Additionally, we investigated the apoptosis induced by CPM NPs under various treatments. As shown in Figure 3B, the apoptosis rates in the CPM, CPM + US, and CPM + L groups were 17.4%, 27.4%, and 42.3%, respectively. Under the application of both laser and ultrasound irradiation, the CPM + US + L group showed the highest apoptosis rate (53.5%). These results are in agreement with those of the MTT and living/dead assays. The above results demonstrated the superior antitumor effect of CPM + US + L. Subsequently, a series of experiments were conducted to elucidate the therapeutic mechanism. Alleviating hypoxia in the tumor microenvironment is critical for tumor therapy. Therefore, we firstly detected the effect of CPM NPs for hypoxia remission in cells. Red fluorescence indicates cell hypoxia. As revealed in Supplementary Figure S4, almost no red fluorescence was exhibited in the normal environment with or without CPM NPs. Under hypoxia environment, the obvious red fluorescence signal appears without CPM NPs. In contrast, the CPM-treated cells only exhibited faint red fluorescence, proving the effective hypoxia remission. The generation of ROS in 4T1 cells was evaluated using a DCFH-DA probe, with green fluorescence as the ROS indicator. CPM-treated cells showed weak fluorescence, which may be ascribed to the ROS production during MnO_2_-induced CDT and mitochondrial Ca^2+^ overload (Wang et al., 2019). When either laser or ultrasound irradiation was applied, CPM-treated cells showed stronger fluorescence intensity (Figure 3C), suggesting that both laser irradiation and ultrasound promoted ROS production. This result may be caused by ultrasound-enhanced mitochondrial Ca^2+^ overload and laser-promoted CDT (Zhang et al., 2021). As anticipated, the CPM + US + L group showed the strongest green fluorescence compared to the CPM + US and CPM + L groups, suggesting the superiority of the combination therapy. Mitochondrial Ca^2+^ overload can damage the mitochondria, triggering a decrease in the mitochondrial membrane potential. We further determined the degree of mitochondrial damage after various treatments using JC-1 (mitochondrial membrane potential probe). J-aggregates with red fluorescence and monomers with green fluorescence were found in normal and damaged mitochondrial membranes, respectively. As shown in Figure 3D, weak green fluorescence appeared in the cells of the CPM group, confirming slight mitochondrial damage. The green fluorescence signal was enhanced under laser and ultrasound stimulation, indicating that the application of US and laser increased mitochondrial damage. As expected, the cells in the CPM + US + L group showed the strongest green fluorescence signal, suggesting that the combination of laser irradiation and ultrasound resulted in the most severe mitochondrial damage. In addition to decreased mitochondrial membrane potential, mitochondrial swelling is an important indicator of mitochondrial damage. Therefore, we performed a mitochondrial swelling assay to verify the mitochondrial damage. Normal mitochondria show strong ultraviolet-visible (UV-vis) absorption at 540 nm, whereas the absorbance at 540 nm decreases when mitochondria are damaged. As shown in Figure 3E, the absorbance values of the cells at 540 nm were significantly decreased after treatment with laser irradiation or ultrasound compared to the PBS and CPM groups, indicating that both laser irradiation and ultrasound can induce strong mitochondrial swelling. As anticipated, the CPM + US + L group exhibited the most obvious mitochondrial swelling, which was consistent with the results of the mitochondrial membrane potential assay. Ca^2+^ is considered a key factor in the regulation of mitochondrial activity (Giorgi et al., 2018; Nemani et al., 2018; Rossi et al., 2019). This inspired us to explore whether mitochondrial damage was related to Ca^2+^ overload. First, we used FITC-labeled CPM NPs and red Lyso-Tracker to investigate the internalization and lysosomal escape of CPM NPs in 4T1 cells. As indicated in Figure 4A, the green fluorescence intensity of CPM-FITC gradually increased with prolonged co-culture time of CPM-FITC with 4T1 cells. Notably, when CPM-FITC was co-incubated with 4T1 cells for 4 h, the green fluorescence of CPM-FITC separated from the lysosomal red fluorescence was clearly observed unlike for those at 0.5 and 1 h. The co-localization rate of CPM-FITC fluorescence and lysosomal fluorescence was calculated using ImageJ analysis. As shown in Figure 4C, the colocalization rate of CPM-FITC fluorescence and lysosomal fluorescence reached 77% after 1 h incubation, but subsequently decreased to 22% after 4 h of incubation, confirming that CPM NPs underwent effective lysosomal escape. We then explored the internalization and lysosomal escape of 4T1 cells under various treatments after co-culture with CPM-FITC for 4 h. Compared to the CPM group, the green fluorescence of 4T1 cells was significantly enhanced after laser or ultrasound irradiation (Figure 4B), verifying that laser and ultrasound irradiation promoted the cellular uptake of CPM NPs. This is attributed to the higher cell permeability caused by the photothermal effect and ultrasonic vibration, which facilitates the entry of nanoparticles into cells (Hu et al., 2020).

In addition, most of the separated green fluorescence was observed in the CPM + US + L group. As shown in Figure 4D, the fluorescence colocalization rates of CPM, CPM + US, CPM + L, and CPM + US + L groups were calculated to be 25%, 19%, 15%, and 10%, respectively. Notably, red fluorescence was significantly reduced in the CPM + US, CPM + L, and CPM + US + L groups, indicating strong lysosomal damage caused by the release of CPM NPs into the cytoplasm (Weyergang et al., 2014; Huang et al., 2019). These results demonstrated that laser and ultrasound irradiation accelerated lysosomal escape, and the combination treatment further promotes this process. Then, we detected the Ca^2+^ uptake by CPM NPs after co-culture with 4T1 cells for 4 h. The green fluorescence of Fluo-4 AM was used as the Ca^2+^ probe. The intracellular Ca^2+^ fluorescence intensity increased in a time-dependent manner in 4T1 cells (Figures 4E,G). To study the effects of laser and ultrasound irradiation on intracellular Ca^2+^ content, we treated 4T1 cells after culturing with CPM NPs for 4 h. As shown in Figure 4F, both the CPM + US and CPM + L groups exhibited brighter green fluorescence than the CPM group, proving that laser and ultrasound irradiation facilitated Ca^2+^ internalization by 4T1 cells. The strongest Ca^2+^ fluorescence intensity in the CPM + US + L group confirmed the superiority of the combined laser and ultrasound treatment for Ca^2+^ uptake. Moreover, quantitative analysis of the fluorescence intensity of Ca^2+^ also proved this conclusion (Figure 4H). The above results indicate that CPM NPs under laser and ultrasound irradiation effectively increase intracellular Ca^2+^ content, resulting in Ca^2+^ overload and thereby inducing mitochondrial damage.

**FIGURE 5:**
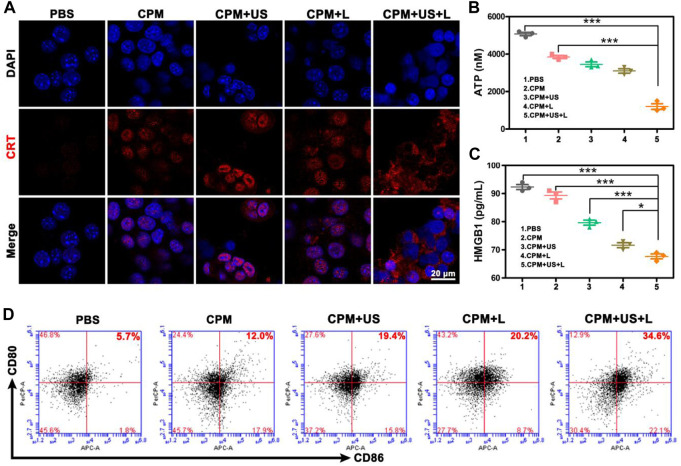
**(A)** CRT exposure. **(B)** Intracellular ATP and **(C)** HMGB1 release from 4T1 cells after various treatments. Data are means ± SD, *p < 0.05, **p < 0.01, and ***p < 0.001. **(D)** Flow cytometry analysis of the expression of CD80^+^/CD86^+^ DC cells (markers for maturation) on the surface of DCs after various treatments.

To assess the antitumor immune response to CPM NPs under various treatments, we explored ICD. The release of DAMPs from tumor cells is an important step in triggering ICD in tumor immunotherapy. During ICD, DAMPs, including CRT, ATP, and HMGB1, can be exposed to the membrane surface of tumor cells to facilitate DC maturation and stimulate optimal antigen presentation. Confocal microscopy was used to observe the level of CRT secretion on the surface of 4T1 cells after treatment, and the levels of intracellular ATP and HMGB1 release were determined using the ATP and HMGB1 ELISA kits, respectively. We found that CRT expression on the cell surface of the CPM group was enhanced compared to that in the PBS group, which may be caused by the Ca^2+^-and Mn^2+^-evoked ICD (Figure 5A). When laser or ultrasound irradiation was applied, a higher CRT exposure was observed. As expected, the CPM + US + L group showed the highest CRT exposure, demonstrating that the laser and ultrasound irradiation promoted immune responses. HMGB1 and ATP have been identified as other ICD biomarkers in 4T1 cells. As illustrated in Figures 5B,C, the intracellular release of HMGB1 and ATP significantly decreased in the CPM + US + L, CPM + US, and CPM + L groups compared to the PBS and CPM groups. Among these, CPM + US + L exhibited the most significant decrease. These results confirmed that CPM + US and CPM + L can trigger an effective ICD that was further promoted by the combination of laser and ultrasound irradiation. Numerous studies have demonstrated that DAMPs released from necrotic or apoptotic tumor cells can stimulate DCs maturation. DCs maturation was measured using flow cytometry. The mean percentage of mature DCs in the CPM + US + L group (34.6%) was significantly higher than that in the PBS (5.7%), CPM (12.0%), CPM + US (19.4%), and CPM + L (20.2%) groups (Figure 5D). These results verified that laser and ultrasound irradiation with CPM NPs can effectively induce ICD and promote DC maturation, thereby activating the antitumor immune response.

### *In vivo* synergistic photothermal therapy and immune therapy

**FIGURE 6:**
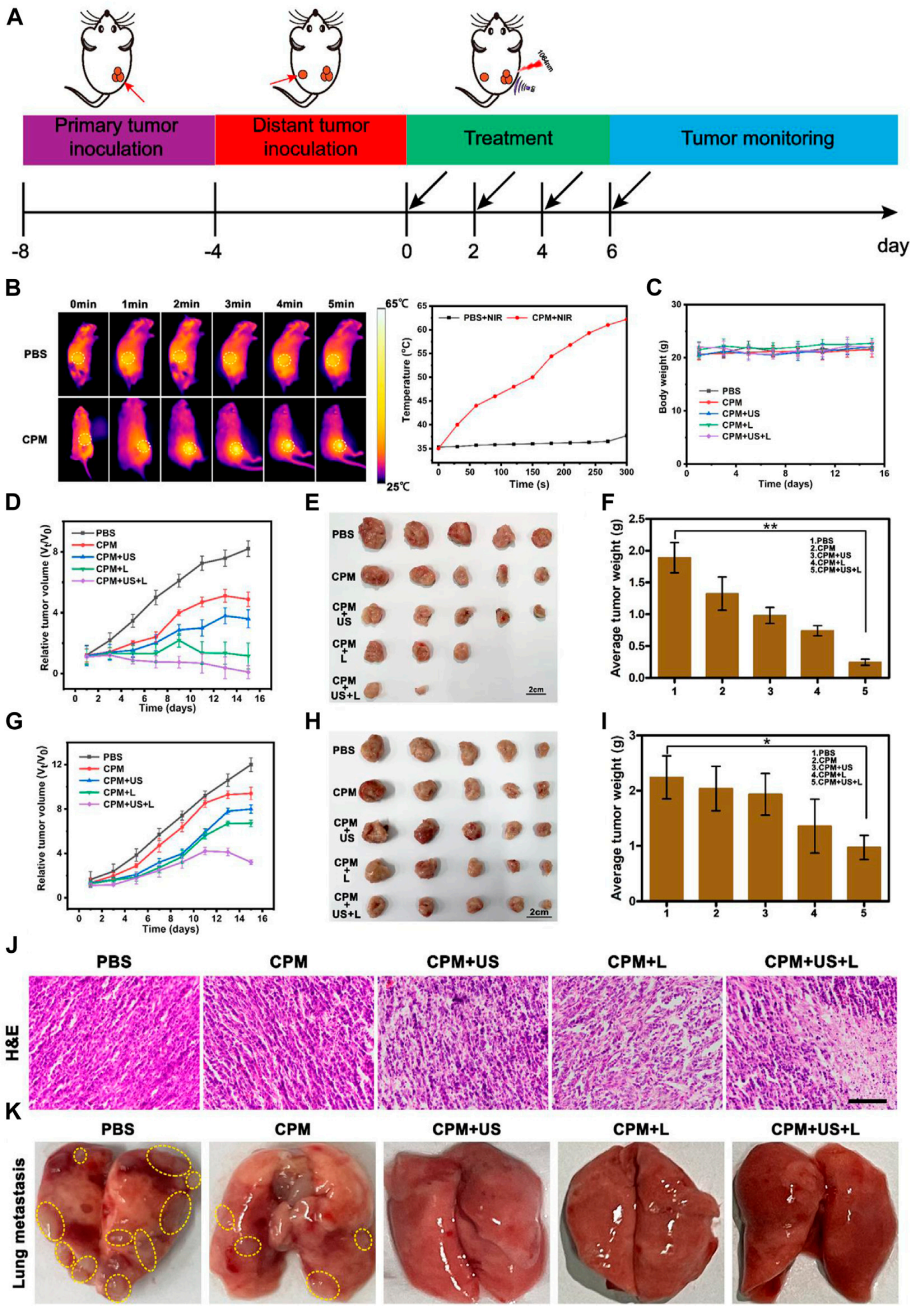
**(A)** Schematic illustration of the animal tumor model and treatment process. **(B)** Infrared thermographic images of mice in PBS + NIR and CPM + NIR groups and the corresponding temperatures curves of mice. (1,064 nm, 1 W cm^−2^, 5 min). **(C)** Average body weight curves during treatment. **(D)** Relative tumor volumes-time curves of 4T1 primary tumors in mice after various treatments. **(E)** Representative tumor pictures of 4T1 primary tumor and **(F)** average tumor weights obtained on the 15 th day. **(G)** Relative tumor volumes-time curve of 4T1 distant tumors in mice after various treatments. **(H)** Representative tumor pictures of 4T1 distant tumor and **(I)** average tumor weights obtained on the 15 th day. Data are means ± SD, *p < 0.05. **(J)** H and E staining images of primary tumors tissues. (Scale bar: 100 μm). **(K)** Representative photographs of lung tissues after various treatments.

Tumor therapy *in vivo* strictly followed the experimental scheme (Figure 6A). First, the therapeutic efficiency of the treatment system for primary and distant tumors was assessed simultaneously using a bilateral 4T1 tumor model. Different numbers of 4T1 cells (2 × 10^6^ cells per mouse and 1 × 10^6^ cells per mouse) were inoculated into both the right (defined as the primary tumor) and left (defined as the distant tumor) flank regions of BALB/c mice on days −8 and −4, respectively. CPM NPs (100 μl, 1 mg ml^−1^) were injected into the primary tumor region of BALB/c mice prior to each irradiation of the BALB/c primary tumors with 1,064 nm laser (1 W cm^−2^, 5 min) or US (1.0 MHz, 20% duty cycle, 2 W cm^−2^, 2 min). First, the photothermal effect of the laser-irradiated BALB/c mice was assessed using an infrared thermal imaging camera. After the injection of PBS or CPM NPs, mouse tumor areas were irradiated with a 1,064 nm laser (1 W cm^−2^), and temperature changes were recorded every 30 s using an infrared camera. Photothermal photographs of the mice showed that the CPM group exhibited time-dependent heating. The temperature of the CPM group increased from 35 to 62.2°C after 5 min of laser irradiation, showing a strong photothermal performance (Figure 6B). By contrast, the temperature for the PBS group only increased by 2.4°C within 5 min of laser exposure. Body weight changes (Figure 6C) and tumor volumes of each mouse were measured every 2 days. The relative volume curves of the tumors (including primary and distant tumors) were plotted according to the records. After 15 days of treatment, the mice were euthanized by dislocation of cervical vertebra and the primary and distant tumors were imaged and weighed. Unlike the malignant proliferation of 4T1 cells in the PBS group, the CPM + US and CPM + L groups showed significant inhibitory effects on tumor growth; notably, the inhibition of tumor growth was most pronounced in the CPM + US + L group (Figure 6D). The tumor images and weights showed the same trend (Figures 6E,F). These results suggest that the combination of photothermal therapy and immunity can significantly inhibit tumor growth, which is consistent with its outstanding antitumor effect *in vitro*. As expected, the growth of distant tumors in mice exhibited the same trend as the growth of the primary tumor. Notably, the CPM + US + L group displayed obvious inhibition compared to the other groups (Figures 6G–I). Histological analysis of the primary tumor was performed to detect tissue damage. As illustrated in Figure 6J, after different treatments, different degrees of apoptosis in tumor cells with shrunken and broken nuclei were observed. The CPM + US and CPM + L groups exhibited more severe apoptosis than the CPM and PBS groups. It is important to note that CPM + US + L showed the greatest tumor cell damage, further confirming the antitumor efficacy of the different treatments.

### *In vivo* anti-lung metastasis ability and immunity of synergistic photothermal therapy and immune therapy

To comprehensively evaluate the antitumor ability of CPM, we also investigated the anti-lung metastasis ability of CPM *in vivo*. As shown in Figure 6K, the lungs of the mice in the PBS group exhibited a large number of pulmonary nodules that were significantly reduced in the CPM group. Additionally, the lung surfaces of the mice in the CPM + US, CPM + L, and CPM + US + L groups were flat, further demonstrating the superior antitumor metastasis ability of this therapeutic platform.

**FIGURE 7:**
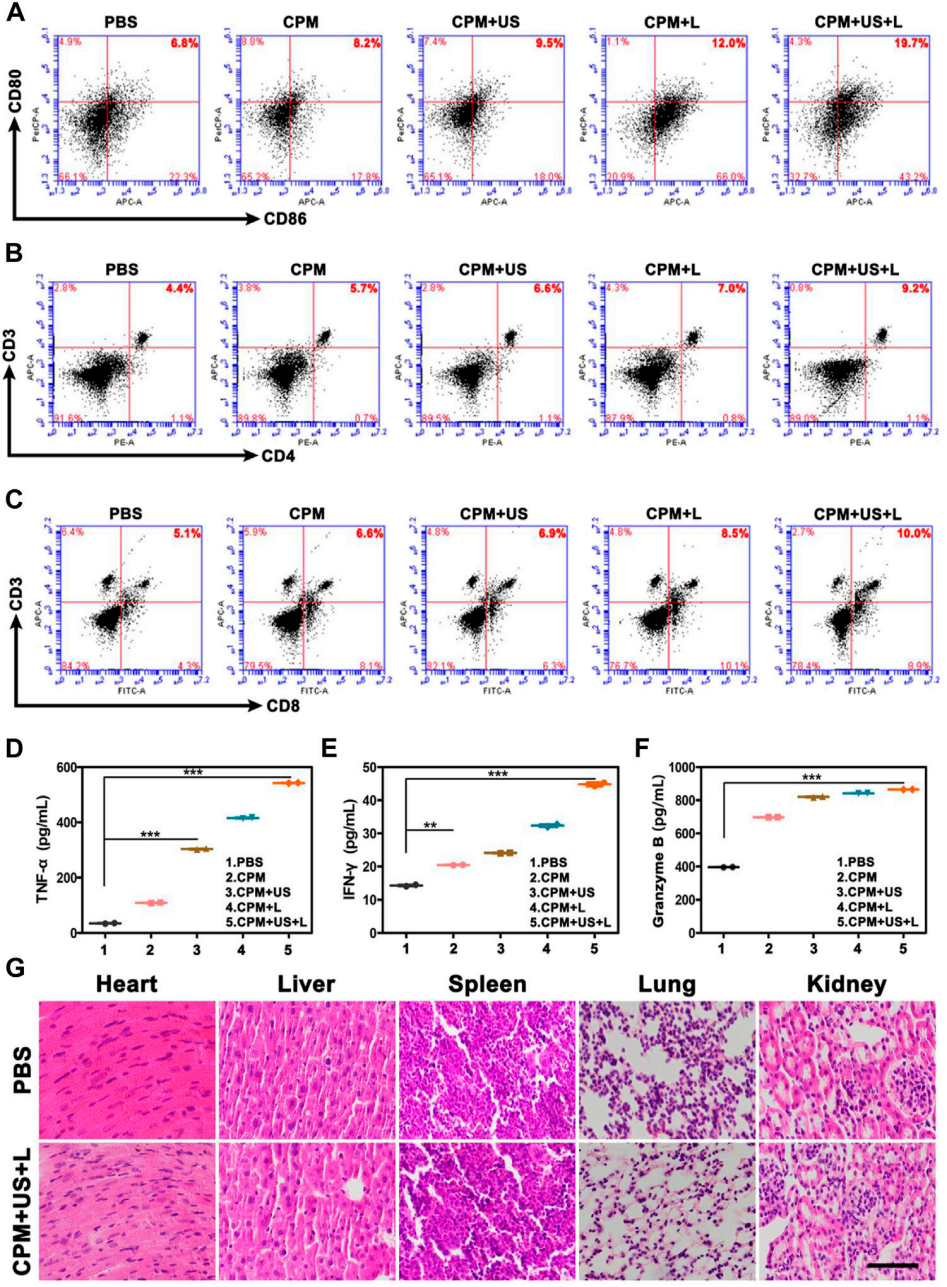
Representative flow cytometry profiles of **(A)** CD80^+^/CD86^+^ DC cells after indicated procedures in the lymph nodes, **(B)** CD3^+^/CD4^+^ T cells in the spleen and **(C)** CD3^+^/CD8^+^ T cells in the spleen. **(D–F)** The ELISA analysis of intraserous cytokine levels of TNF-α, IFN-γ, and Granzyme B after different treatments on day 15. **(G)** H and E-stained images of major organs of BALB/c mice in various groups. (Scale bar: 100 μm).

After the ability of CPM NPs to induce ICD and facilitate DC cell maturation was verified *in vitro*, we further investigated the antitumor immune responses of CPM NPs after various treatments *in vivo*. Flow cytometry was used to determine the DCs maturation (CD11c^+^/CD80^+^/CD86^+^) levels in the draining lymph nodes. As expected, the highest secretion level of DCs maturation (19.7%) was observed in the CPM + US + L group compared to the CPM (8.2%), CPM + US (9.5%), and CPM + L (12%) groups (Figure 7A), indicating that the combined laser and ultrasound therapy with CPM NPs effectively induced DCs maturation and improved the immune response *in vivo*. In addition, CD4^+^ and CD8^+^ T lymphocytes in the spleen were detected using flow cytometry. As shown in Figures 7B,C, the CPM + US + L group exhibited the strongest T cell-mediated immune response (CD4^+^/CD8^+^ T cells: 9.2% and 10%) compared to the PBS (4.4% and 5.1%), CPM (5.7% and 6.6%), CPM + US (6.6% and 6.9%), and CPM + L (7.0% and 8.5%) groups, suggesting that combined treatment with CPM NPs promotes CD4^+^ and CD8^+^ T lymphocyte proliferation and infiltration. Subsequently, immune-mediated tumor killing was assessed by determining the levels of typical immune-related pro-inflammatory cytokines secreted by mature DC. The levels of cytokines TNF-α, IFN-γ, and granzyme B in mouse serum were measured by enzyme-linked immunosorbent assay. As illustrated in Figures 7D–F, the highest cytokine levels were found in the CPM + US + L group. Furthermore, the immune cell response against the tumor was also evaluated by immunohistochemistry analysis of tumor sections from mice. As expected, the highest proportion of CD4^+^ and CD8^+^ T cell infiltration in the tumor tissues were found in the CPM + US + L group (Supplementary Figure S4), suggesting that the combination of laser and ultrasound treatment could further promote the infiltration and proliferation of CD4^+^ and CD8^+^ T cells. These results indicated that the regimen of the combination photothermal and immune tumor therapy significantly enhanced the antitumor immune response.

### Systemic toxicity evaluation

**FIGURE 8:**
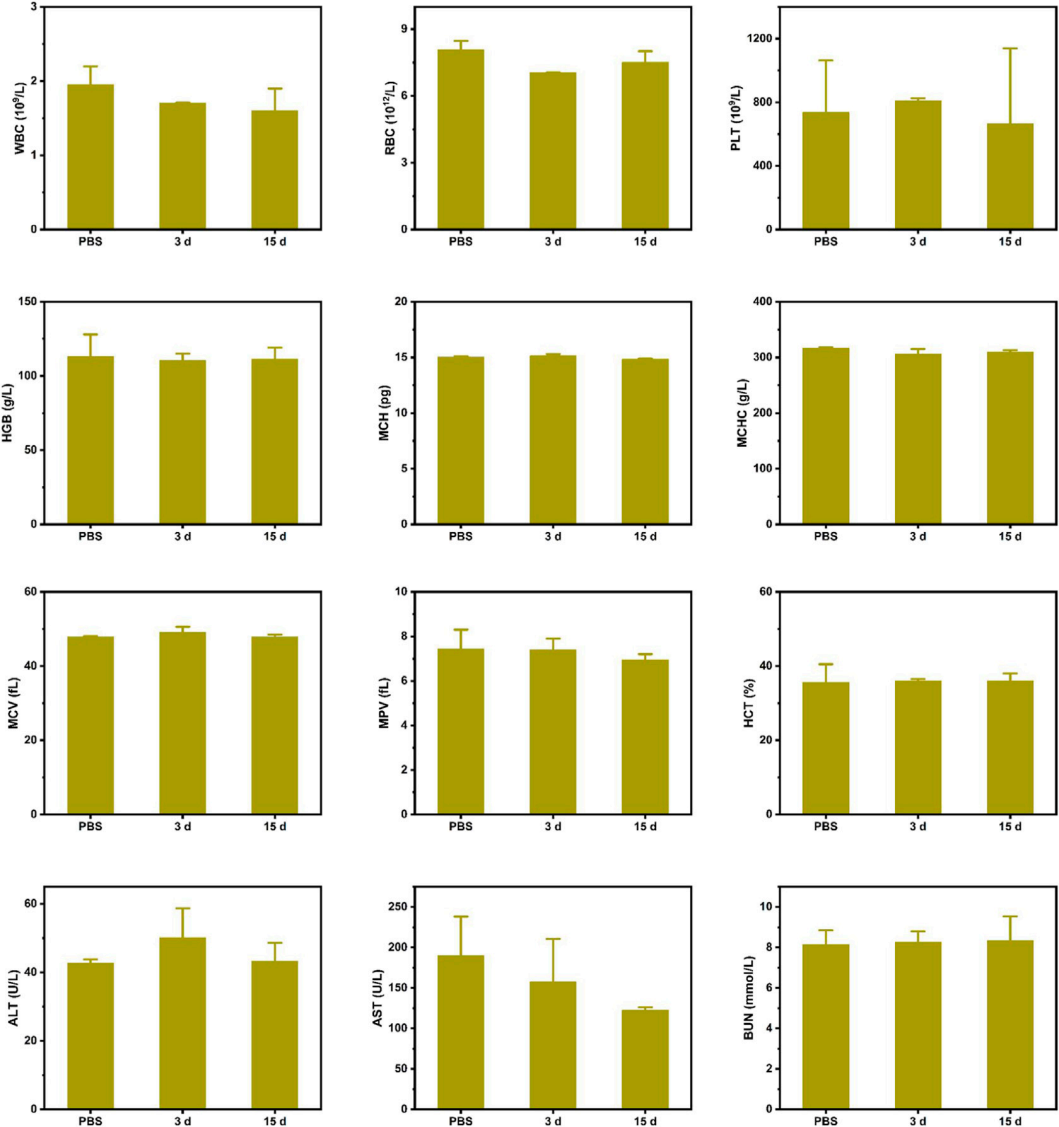
Major blood routine indexes, liver and kidney function indexes of mice after various treatments.

Furthermore, to assess the biosafety of CPM, we performed histological analysis of major organs, including the heart, liver, spleen, lungs, and kidneys. As shown in Figure 7G, H and E-stained images of organ sections from the different treatment groups showed negligible abnormalities. Furthermore, the mice did not experience significant weight loss during the treatment period (Figure 6C), indicating the excellent biocompatibility of CPM NPs and the safety of the combined treatment procedure. Moreover, the functional indicators of the blood samples in the CPM + US + L group were tested to assess the potential toxicity of CPM *in vivo*. As shown in Figure 8, compared to the PBS group, the CPM + US + L group showed negligible abnormalities in the relevant functional indicators on day 3 and at the end of treatment. The routine blood indices in the CPM + US + L group were found to be normal compared to those in the PBS group, indicating that no significant systemic infection or inflammation appeared during the entire evaluation period. The measured parameters, including alanine aminotransferase (ALT), aspartate aminotransferase (AST), and urea nitrogen (BUN), were within the normal range, indicating that the nanoparticles exhibited little toxicity to the liver. These results demonstrated the excellent compatibility and negligible adverse effects of the CPM + US + L treatment on the organism.

## Conclusion

In summary, a combined photothermal and immune tumor therapy platform was fabricated based on dual-gas nanogenerators (CPM NPs). The CPM NPs underwent responsive degradation in the tumor, resulting in the generation of Ca^2+^, Mn^2+^, CO_2_, and O_2_. Ca^2+^ and Mn^2+^ act as immune adjuvants that trigger ICD for immunotherapy. The relieved tumor hypoxia and cancer cell membrane rupture caused by sudden burst of bubbles (CO_2_ and O_2_) all contributed to ICD. The application of laser and ultrasound promoted the Ca^2+^ overload in mitochondria and explosion of CO_2_ and O_2_ bubbles by photothermal effects and ultrasonic waves thus improved immunotherapy effect. Superior PTT and immune synergistic therapeutic capabilities were demonstrated in both *in vitro* and *in vivo* experiments. Thus, this strategy based on CPM NPs dual-gas nano-generators is promising for future clinical cancer treatment.

## Data Availability

The original contributions presented in the study are included in the article/Supplementary Material, further inquiries can be directed to the corresponding authors.
