# Supplementary material for: Ultrasound and laser-promoted dual-gas nano-generator for combined photothermal and immune tumor therapy
Source: Front Bioeng Biotechnol. 2022 Sep 13;10:1005520. doi: 10.3389/fbioe.2022.1005520 (PMC9513372; doi:10.3389/fbioe.2022.1005520)
Supplement: Supplementary file 1 [file DataSheet1.PDF]

Supplementary Material

Ultrasound and laser-promoted dual-gas nano-generator for combined photothermal and immune tumor therapy

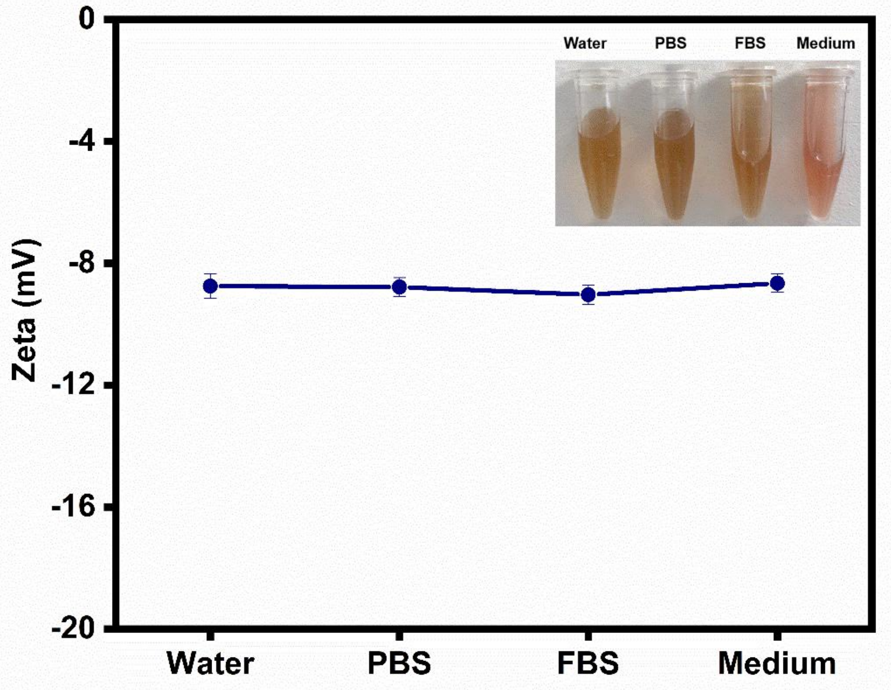

Supplementary Figure 1. The average zeta potentials and digital photograph of CPM NPs (1 mg mL^{-1}) dispersed in Water, PBS (pH 7.4), FBS, and Medium for 1 week.

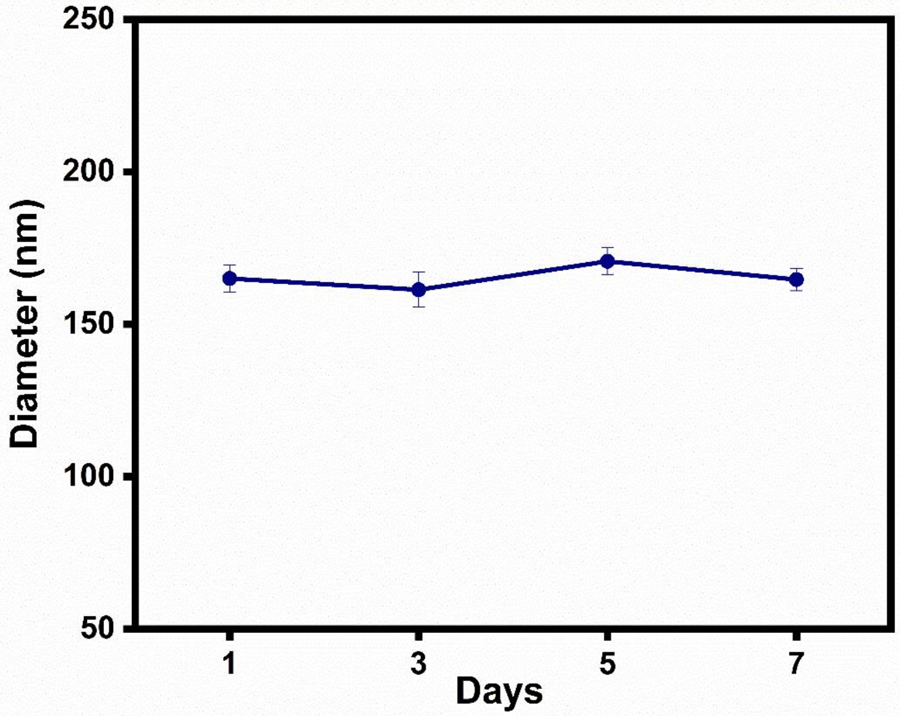

Supplementary Figure 2. Hydrodynamic diameter distribution of CPM NPs (1 mg mL^{-1}) after immersion in water at different time point.

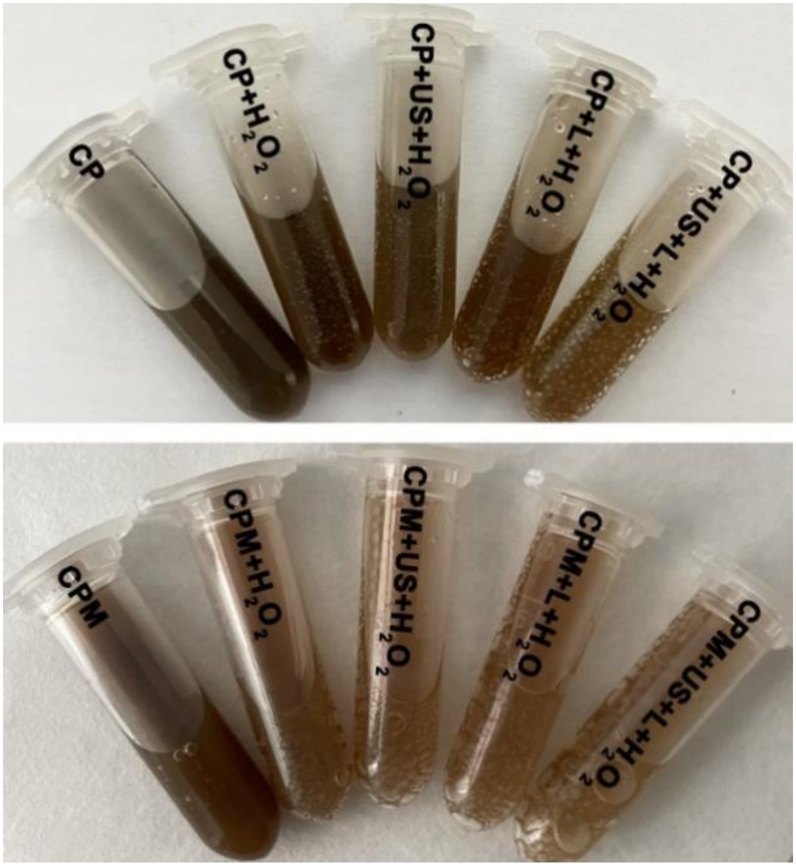

Supplementary Figure 3. Digital photograph of CP NPs (5 mg mL^{-1}) and CPM NPs (5 mg mL^{-1}) incubated with H_2O_2 (100 μM) for 1 min after various treatments.

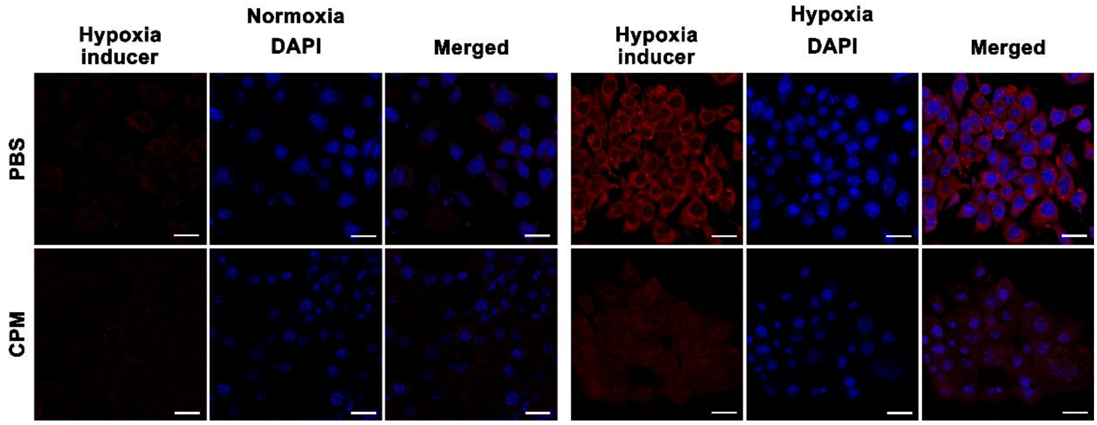

Supplementary Figure 4. The hypoxia probes detection of the oxygen production capacity of CPM NPs in 4T1 cells (Scale bar: 20 μm).

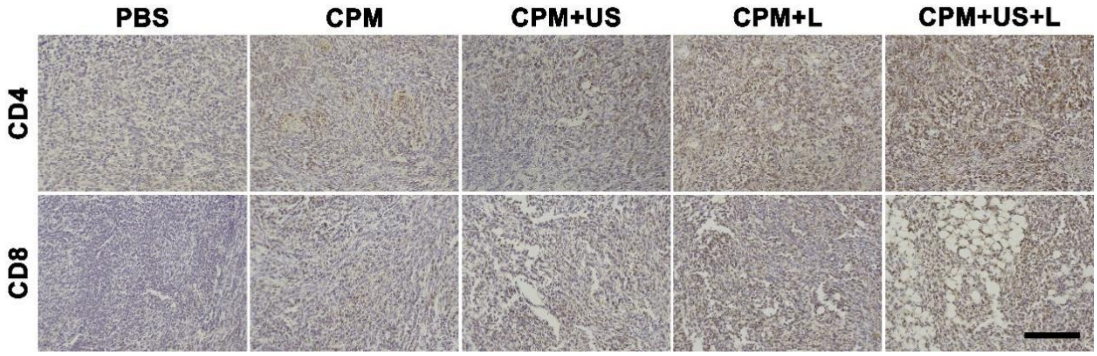

Supplementary Figure 5. $CD4^{+}$ and $CD8^{+}$ immune-histochemical analysis of the dissected tumors of various groups (Scale bar: 50 μm).
